# Research Status and Development Trends of Joining Technologies for Ceramic Matrix Composites

**DOI:** 10.3390/ma18040871

**Published:** 2025-02-17

**Authors:** Biao Chen, Hang Sun, Yuchen Ye, Chunming Ji, Shidong Pan, Bing Wang

**Affiliations:** 1 China Helicopter Research and Development Institute, Jingdezhen 333001, China; 2 National Key Laboratory of Science and Technology for Advanced Composites in Special Environments, Harbin Institute of Technology, Harbin 150001, China

**Keywords:** ceramic matrix composites, joining technology, mechanical property, application prospects

## Abstract

Ceramic matrix composites (CMCs) are composite materials made by using structural ceramics as matrix and reinforcing components such as high-strength fibers, whiskers, or particles. These materials are combined in a specific way to achieve a composite structure. With their excellent properties, including high specific strength, high specific stiffness, good thermal stability, oxidation resistance, and corrosion resistance, CMCs are widely used in the aerospace, automotive, energy, defense, and bio-medical fields. However, large and complex-shaped ceramic matrix composite parts are greatly influenced by factors such as the molding process, preparation costs, and consistency of quality, which makes the joining technology for CMCs increasingly important and a key trend for future development. However, due to the anisotropic nature of CMCs, the design of structural components varies, with different properties in different directions. Additionally, the chemical compatibility and physical matching between dissimilar materials in the joining process lead to much more complex joint design and strength analysis compared to traditional materials. This paper categorizes the joining technologies for CMCs into mechanical joining, bonding, soldering joining, and hybrid joining. Based on different joining techniques, the latest research progress on the joining of CMCs with themselves or with metals is reviewed. The advantages and disadvantages of each joining technology are summarized, and the future development trends of these joining technologies are analyzed. Predicting the performance of joining structures is currently a hot topic and challenge in research. Therefore, the study systematically reviews research combining failure mechanisms of ceramic matrix composite joining structures with finite element simulation techniques. Finally, the paper highlights the breakthroughs achieved in current research, as well as existing challenges, and outlines future research and application directions for ceramic matrix composite joining.

## 1. Introduction

**Figure 1:**
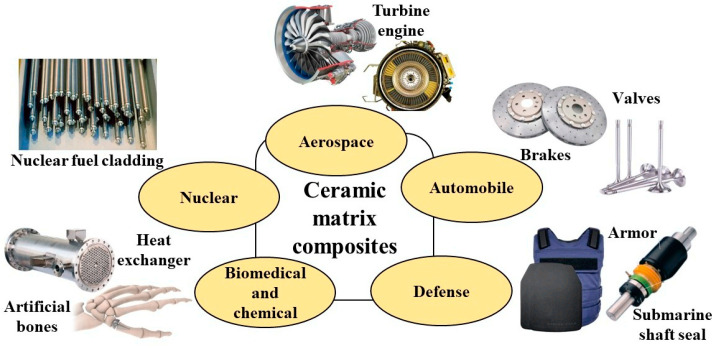
Application of CMCs in various fields [1].

With the rapid development of modern technology, the demand for high-performance materials in fields such as aerospace, energy, and automobiles is increasing day by day. Ceramic matrix composites (CMCs), with outstanding properties such as high specific strength, high specific stiffness, excellent thermal physical stability, oxidation resistance, and corrosion resistance, have shown great application potential in many high-end fields [1]. CMCs are composite materials formed by combining structural ceramics as the matrix with high-strength fibers, whiskers, and particles as the reinforcing materials through certain methods. CMCs can be classified into oxide ceramic matrix composites and non-oxide ceramic matrix composites based on the matrix materials. The oxide matrix materials include alumina (corundum), aluminosilicate (mullite), zirconia (zircon), etc., while the non-oxide matrix materials include silicon carbide (SiC), zirconium carbide (ZrC), boron nitride (BN), aluminum nitride (AlN), etc., among which silicon carbide-based composites are the most widely used and have developed the fastest [2]. CMCs are widely applied in engineering components such as heat exchangers, wear-resistant parts, and thermal protection systems, as shown in Figure 1. Due to their ability to maintain structural stability under high-temperature and high-pressure conditions, ensuring the efficient operation of engines, CMCs are commonly found in the hot-end components of aero engines. Additionally, CMCs can withstand extreme temperature changes and are widely used in the thermal protection structures of spacecraft in the aerospace field to safeguard the safety of internal equipment. In the energy sector, especially in nuclear power plants, ceramic matrix composites are used to manufacture the cladding materials of nuclear reactors, effectively resisting radiation and high-temperature corrosion, ensuring the long-term stable operation of nuclear facilities. CMCs possess the characteristics of high specific strength, low weight and wear resistance, and are widely used in the defense industry, such as in tank reinforced armor, flamethrowers, wear-resistant precision bearings and so on. In the biomedical industry, advanced ceramic matrix composites are used to manufacture various implant materials due to their high biocompatibility, high strength, and durability. Moreover, due to their significant catalytic adsorption properties and high chemical corrosion resistance, CMCs also play a crucial role in the chemical industry [3]. In recent years, with the rapid development of metamaterials [4], CMCs have been extensively studied in the field of metamaterials due to their excellent properties. By designing their microstructure, CMCs can achieve unique mechanical properties, such as negative compressibility, negative stiffness [5], and negative Poisson’s ratio [6], among others. These materials are widely applied in various fields, including energy absorption [7], vibration isolation [8], and deformable structures.

However, although CMCs have numerous outstanding properties, their machinability is poor. Fabricating large-sized components with complex shapes faces many difficulties, which largely limits their applications. Joining technology is a key means to address these issues. It can connect CMCs with themselves or metal materials to form complex structural components, thereby meeting the diverse needs of different engineering fields and fully exploiting their performance advantages. This is of crucial significance for expanding the application scope of CMCs in fields such as aerospace, energy, and the defense industry and promoting the development of related industries [9].

Currently, the joining technologies of CMCs are mainly classified as mechanical joining, bonding, soldering, and hybrid joining [10]. In this paper, according to different joining technologies, the latest research progress in the joining of ceramic matrix composites with themselves or with metals is reviewed, respectively. The joining methods, mechanisms, and results are emphatically introduced. The advantages and disadvantages of various joining technologies are summarized, and the future development trends of each joining technology are analyzed. The research on combining the failure mechanism of ceramic matrix composite joint structures with finite element simulation technology is systematically combed and discussed to achieve performance prediction of joint structures. Finally, the breakthrough progress and remaining problems of current research are discussed, and the future research and application directions of ceramic matrix composite joining are anticipated.

## 2. Mechanical Joining Technology

Mechanical joining, an important category of ceramic matrix composite joining technologies, plays an indispensable role in numerous engineering applications. It mainly joins CMCs with other components through joints like bolts and rivets to form a stable integral structure.

The mechanical joining technology has the following advantages:

1. Robust connection. It is not prone to loosening or failure when subjected to relatively large external forces, providing a reliable joining effect;
2. Relatively simple operation. It does not require complicated chemical or physical reaction processes and is easy to master and implement. Thus, it has high flexibility in on-site construction and assembly processes;
3. Facilitates disassembly and reinstallation. When it comes to equipment maintenance or the need to replace components, it is convenient for inspection and component replacement, reducing maintenance costs and time.

However, mechanical joining also faces the following challenges:

1. Increasing the overall weight of the structure. For some weight-sensitive application scenarios (such as in the aerospace field), this may have an adverse impact and reduce the lightweight advantage of the materials;
2. Damaging the connected parts. During the installation process, it may cause damage to the ceramic matrix composite substrates, affecting the material’s performance and structural integrity;
3. Low economic efficiency. High-quality mechanical joints will lead to increased processing and installation costs, increasing the total cost of the product [11].

### 2.1. Bolt Joining Technology

**Figure 2:**
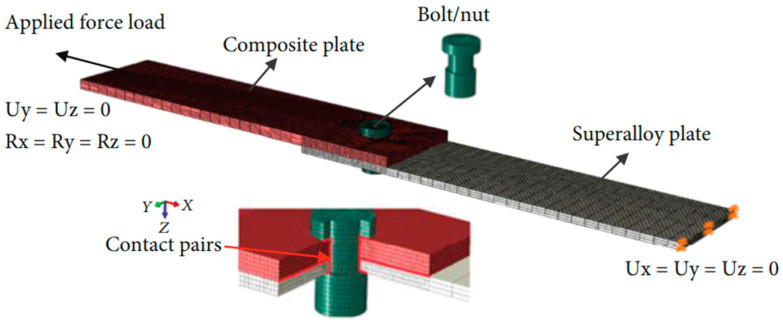
Finite element model and contact pair setup of 2D C/SiC composite—high-temperature alloy bolt joint structure [13].

Bolt joining is a common mechanical joining method for CMCs. It can endure relatively large tensile, shear, and other loads and can apply to structural connections with high load-bearing capacity requirements [12]. However, as CMCs are often used in high-temperature and high-pressure environments, higher demands are imposed on bolted joining structures, which need to be fully considered in design and application. Zhao et al. [13] developed a finite element model, as shown in Figure 2, to simulate the thermal structural behavior and high-temperature tensile performance of a single-lap single-bolt ceramic matrix composite/high-temperature alloy joint through progressive damage analysis. It was found that the thermal expansion mismatch between CMCs and high-temperature alloy materials led to a sharp drop in preload and an unfavorable change in bolt–hole clearance. Temperature had a significant impact on the tensile performance and damage propagation of the joint structure. Zhao et al. [14] established a finite element model and used progressive damage analysis to study the influence of geometric parameters of double-bolt joints on failure load and damage mechanism at 750 °C. It was found that edge distance significantly affected the high-temperature bearing capacity of double-bolt joints, showing a trend of first increasing and then decreasing. A larger edge distance would change the stress concentration at the hole edges of ceramic matrix composite plates and bolt tightening efficiency. Plate edge distance and center-to-center spacing of double bolts had relatively little impact on the failure load of the connection structure at 750 °C. An increase in plate edge distance would slightly increase connection stiffness and enhance joint strength under the same failure load. When the center-to-center spacing of double bolts was different, the failure modes of CMCs were different, and multiple failure modes interact to cause changes in failure loads.

### 2.2. Rivet Joining Technology

**Figure 3:**
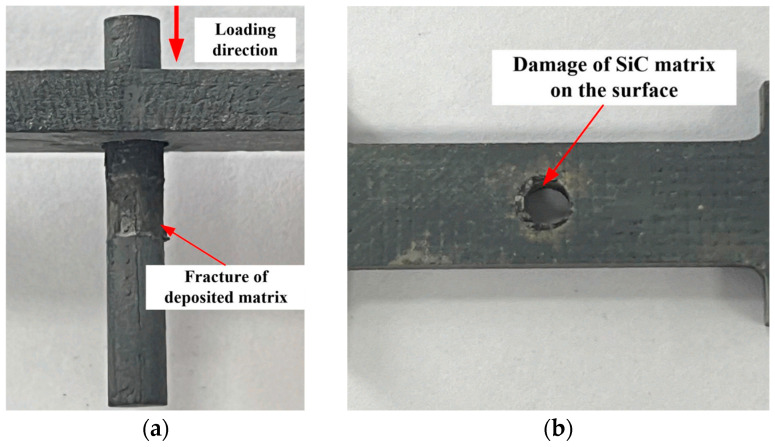
Failure modes of C/SiC riveted joints: (**a**) Specimen with rivets; (**b**) specimen with rivets fully ejected [15].

In the joining of CMCs in specific fields, the riveting process is often employed. However, as the force transmission between rivets and hole walls mainly depends on friction and mechanical interlocking, its joining strength is relatively low. When subjected to large tensile or shear forces, it is prone to shear deformation or pull-out. Xu et al. [15] investigated the failure mechanism of C/SiC riveted joints under thermo-mechanical coupling loads. It was revealed that the predominant failure mode of C/SiC riveted joints was a brittle fracture of the interface layer between the rivets and the plates, as depicted in Figure 3. During the failure process, due to the friction resulting from interference fit, the progressive damage was mainly concentrated on the surface of the C/SiC composite material. As the environmental temperature increased to 800 °C, the out-of-plane ultimate strength of the C/SiC riveted joints decreased. Xu et al. [16] studied the impact of temperature changes and the residual stress generated by riveting interference fit on the properties of C/SiC composite materials by combining experimental and simulation analysis methods. Owing to the release of residual stress enhancing the interface performance, the fracture cross-sectional morphology transformed from the tortuous pull-out of fibers to neat brittle fracture. At room temperature, the rivets moderately enhanced the load-bearing capacity of C/SiC composite materials. Nevertheless, as the environmental temperature increased, the load-bearing capacity decreased significantly.

### 2.3. Ceramic Matrix Composite Fasteners

**Figure 4:**
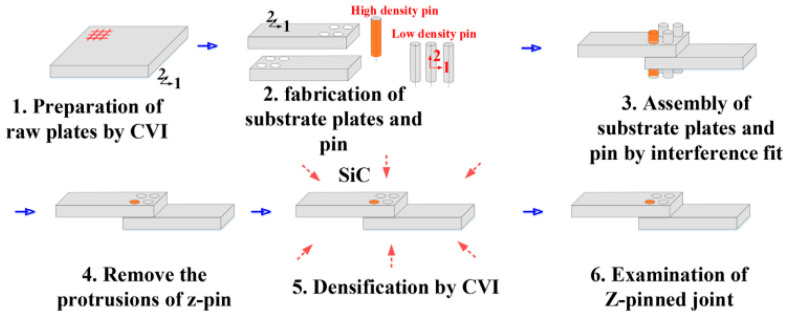
Preparation of a two-dimensional C/SiC composite Z-direction pin joint with four rectangular arrays [19].

**Figure 5:**
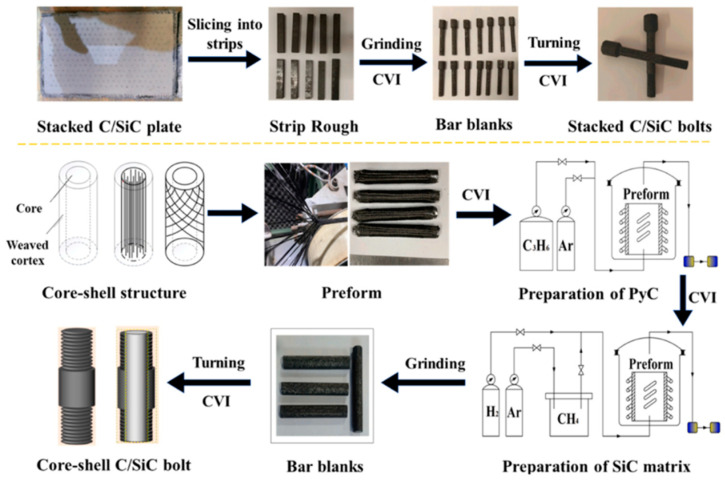
Preparation process of laminated and core–shell C/SiC bolts [20].

The thermal expansion coefficients of traditional metal fasteners and the connected ceramic matrix composite components are mismatched, which leads to discrepancies in deformation between the two under high-temperature conditions, thereby reducing the stability and reliability of the overall connection structure. Additionally, metal fasteners are limited by their operating temperature and are not suitable for connecting CMCs. To meet the requirements of high temperature and weight reduction, ceramic matrix composite fasteners have been developed and implemented in engineering applications, attracting significant attention. However, the density distribution of ceramic matrix composite fasteners is non-uniform, and the pin load distribution is different from that of metal connections. Consequently, the performance of ceramic matrix composite fasteners is notably inferior to that of metal fasteners [17]. Moreover, unlike the linear mechanical behavior of metal fasteners, the pseudo-plastic mechanical behavior of ceramic matrix composite fasteners and the influence of the non-uniform microstructure must be considered. Zhang et al. [18] adopted two-dimensional C/SiC Z-shaped pin joints prepared by chemical vapor infiltration (CVI) and applied axial static loads and cyclic loads to them. It was found that short-term fatigue loading might have a positive impact on joint strength. The cracks generated by fatigue loading led to stress redistribution, which was beneficial for improving the strength. The Z-type rivet joint demonstrates excellent fatigue resistance under cyclic loading, particularly at static limit stress levels ranging from 55% to 78%. The joint can withstand up to 10^5^ fatigue cycles without failure. During the initial stages of fatigue loading, damage is concentrated within the first few hundred cycles, after which it enters a stable phase. The residual strength increases during the early stages of fatigue cycling, reaching a peak at 5 × 10^4^ cycles, and then starts to decrease. This phenomenon is attributed to crack propagation and the redistribution of local stresses during the fatigue loading process, leading to an initial strengthening of the joint. Acoustic emission (AE) analysis further confirms the evolution of fatigue damage, indicating that after fatigue loading, damage primarily occurs when the stress exceeds the historical maximum fatigue stress upon reloading. Sun et al. [19] experimentally prepared two-dimensional C/SiC four-rivet rectangular array Z-shaped rivet joints of rivets, as shown in Figure 4. It was found that due to the uneven force on each rivet, there were different proportional relationships between the tensile strength of the two-dimensional C/SiC four-rivet square riveting unit and that of a single rivet before and after oxidation. Oxidation reduced the degree of unevenness in the rivet load distribution, making the longitudinal rivet load distribution uniform. After oxidation, the bottom rivets bore the load first, and when the limit was reached, the top rivets began to bear the load. The longitudinal rivets could transfer loads, and the failure modes of fiber bundles in different regions inside the rivets were different. Ma et al. [20] prepared a new type of core–shell structure C/SiC bolts by the CVI method, as shown in Figure 5. Compared with laminated structure bolts, the core–shell structure design made the thread gear profile smooth and complete, improving the thread meshing accuracy and the bearing area. The change in fiber orientation transformed the stress state of the fibers and increased the fiber volume fraction, thereby enhancing the thread pull-off strength. The strength of a single thread tooth increased by 17.96%. Notably, 0° unidirectional C/SiC composite materials and ZrO_2_ ceramics increased the shear strength of the core–shell bolts by 45.29% and 46.88%, respectively. However, due to the clearance, it was difficult for the shell layer and the rod to bear the load simultaneously, and the tensile fracture strength of the bolt rod only increased by 2.96%. Yuan et al. [21] employed infrared thermal imaging and acoustic emission monitoring technologies to perform in situ evaluations on the damage process of C/SiC composite bolts under tensile loads. By analyzing the force-displacement curves, infrared signals, acoustic emission signals, and fracture morphologies, the damage process was divided into three stages. The effectiveness of the monitoring methods was verified, providing a reference for further research on the failure mechanisms of this material under different loads. Ma et al. [22] fabricated laminated three-dimensional needled (3DN) C/SiC countersunk bolts with in situ-toughened SiC whiskers. The SiC whiskers enhance the toughness of the SiC matrix via crack bridging and deflection, improve the load transfer between the laminated SiC matrix and carbon fibers, and thereby increase the tensile strength of the bolts. Nevertheless, the shear strength of the bolts is not enhanced. The introduction of SiC whiskers significantly improves the fatigue performance of bolts, particularly at static limit loads ranging from 50% to 75%, where the bolts can withstand up to 10^6^ fatigue cycles without failure. During the initial stages of fatigue loading, damage is concentrated in the first few hundred cycles, after which it enters a stable phase. The SiC whiskers enhance the toughness of the SiC matrix through crack bridging and deflection effects, thereby improving the fatigue resistance of the bolts. Furthermore, after fatigue loading, the bolts show less damage upon reloading, indicating that the SiC whiskers play a key role in suppressing crack propagation. Zhou et al. [23] prepared plain weave SiC/SiC Z-pin joints by the chemical vapor infiltration method and studied their oxidation behavior and failure mechanism. It was found that the oxidation of the pin joints was uneven, and the degree of oxidation decreased from both ends of the pin to the central area. It showed different characteristics under different oxidation conditions, which affected the fiber–matrix interface and then affected the fracture morphology and failure mechanism of the specimen.

## 3. Bonding Technology

Bonding, an important joining technology for CMCs, employs adhesives to create bonding forces between two or more surfaces to be joined, thus achieving the connection purpose [24]. This connection method can join components with complex shapes and enable the joint between different materials.

It has the following advantages:

1. Superior sealing performance. The bonding connection can effectively prevent the leakage of media such as gases and liquids, making it suitable for application scenarios with high sealing requirements;
2. Low preparation temperature. Bonding operations are typically conducted at relatively low temperatures, exerting minimal thermal influence on CMCs and reducing material property changes and residual stresses caused by high temperatures;
3. Relatively simple process. It does not require complex equipment or special process conditions, facilitating large-scale production and on-site construction. Additionally, during the connection process, it does not damage the connected components and has a relatively minor impact on the properties of the connected materials.

However, the bonding technique also faces the following challenges:

1. Relatively low strength. It may fail when subjected to large external forces, which limits its application in the joint of high-strength structural components;
2. Poor high-temperature resistance. The performance of the adhesive may decline at high temperatures, resulting in a reduction in joint strength. Therefore, the bonding is not suitable for long-term use in high-temperature environments [25].

**Figure 6:**
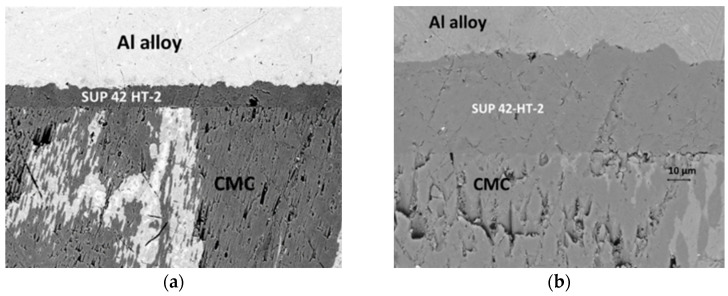
(**a**) Scanning electron microscope (SEM) cross-sectional micrograph of the CMC/EN AW—6082 joint using SUP42 HT-2 (Master Bond, The USA) adhesive and post-heat-treated at 200 °C for 10 min, and (**b**) magnified view of the interface [26].

Valentina et al. [26] employed epoxy resin adhesives to bond CMCs and aluminum alloys. Under the standard process (12 h at room temperature followed by 5 h at 90 °C), the shear strength of the joint was 31.6 ± 7.2 Mpa. For the samples subjected to heat treatment at 200 °C for 10 min, the microscopic morphology of the joint is shown in Figure 6, and the shear strength increased by approximately 21%. Thermal cycling had a minimal impact on the mechanical properties of the connected samples. The salt spray test significantly reduced the mechanical strength of the samples, but there was no statistically significant difference between the test results at 240 h and 720 h. Sandblasting the surface of the aluminum alloy can increase the surface roughness and enhance the bonding strength. Although anodizing treatment can improve the corrosion resistance and wear resistance of the aluminum alloy, it reduces the adhesiveness of the adhesive, resulting in a decrease in shear strength, especially when post-heat treatment is conducted. Yan et al. [27] investigated the influence mechanism of ultrasonic vibration on the bonding joints of CMCs. The proposed hydrodynamic model and capillary rise model can effectively predict the adhesive pressure and penetration ability. It was verified that ultrasonic vibration can increase the maximum hydrodynamic pressure of adhesives by five to seven orders of magnitude compared to the case without vibration, enabling the adhesive to better penetrate the microstructure of the bonding surface and improving the interfacial bonding. The average shear strength of the bonding joints treated with ultrasonic vibration is 15.52 Mpa, while that of the untreated ones is 12.96 Mpa. The ultrasonic treatment increases the joint strength by 19.8% and enhances the mechanical properties of the joints.

**Figure 7:**
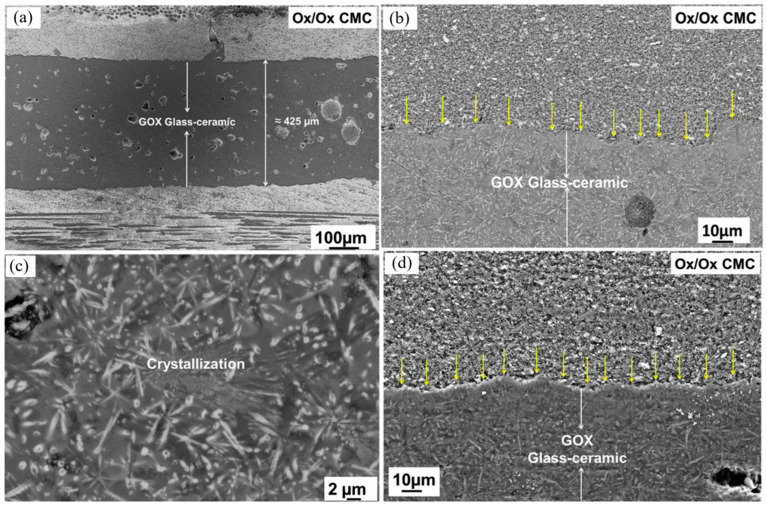
SEM images of the joint and GOX microcrystalline glass crystallization. (**a**) Cross-sectional view of the joint; (**b**) cross-sectional view of the joint in backscattered electron mode (the arrow indicates the bonding interface); (**c**) crystalline phase (bright color) in the glass matrix (dark color); (**d**) cross-sectional view of the joint after thermal aging for 100 h at 850 °C in air (the arrow indicates the bonding interface) [28].

**Figure 8:**
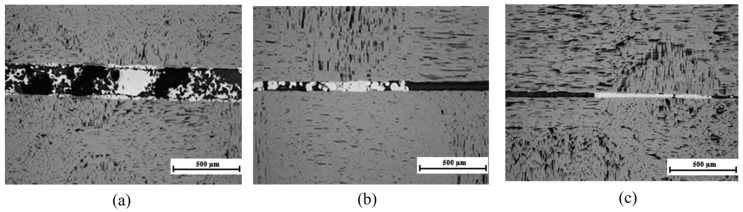
Optical micrographs of the vertical SA-Tyrannohex assembly (⊥-to-⊥) bonded using (**a**) Si-Cr, (**b**) Si-Ti, and (**c**) Si-Hf eutectic phase pastes [32].

To enhance the strength of bonding joints, numerous new technological means have been developed to bond CMCs. Muhammad et al. [28] employed a novel glass–ceramic GOX (SiO_2_-CaO-Al_2_O_3_-MgO-Y_2_O_3_-ZrO_2_) to join CMCs for the first time. Selecting a joining temperature of 1010 °C can ensure maximum crystallization and dense connection while not altering the microstructure and mechanical properties of the composites. The joining process is conducted without pressure in the air, resulting in a continuous and defect-free interface. After 100 h of thermal aging in the air at 850 °C, the joint interface remains continuous and defect-free, indicating stable joining. The microscopic morphology of the joint is shown in Figure 7. In the shear test, delamination of the composites occurs and there is no fracture in the joint area. In the four-point bending test, the bending strength of the glass–ceramic joint is approximately 54% of that of the original composite. Federico et al. [29] successfully fabricated yttrium disilicate-based glass–ceramic materials via reactive viscous flow sintering for joining C/SiC and SiC/SiC composites. After joining, the shear strength was approximately 35 MPa, which was comparable to the interlaminar shear strength of CMCs. The joints demonstrated certain self-healing behaviors, and indentations and cracks could be healed through a two-step annealing process. Carla et al. [30] used two processes to join two different SiC/SiC CMCs with yttrium aluminum silicate (YAS) glass ceramics. The interface adhesion of the joints fabricated by the P2 process was remarkably enhanced, and the porosity of the glass–ceramic layer was decreased. The shear strength of the joints was higher than the interlaminar shear strength of the composite materials, and also much greater than that of the joints produced by the P1 process. Renato et al. [31] put forward a novel approach for joining oxide ceramic matrix composites based on ionotropic gelation. The efficacy of joining Nextel610/alumina–zirconia composites at different processing stages was investigated. The joint in the gel or green state shows excellent results. There is no obvious discontinuity between the joint and the composite plate, and its properties are similar to those of the composite plate matrix. The shear strength of the joint is approximately 8 MPa, which is comparable to the interlaminar shear strength of the composite plate. It exhibits good compatibility when used at high temperatures. Moreover, joints in the green state do not require additional heat treatment. After sintering, voids and cracks exist at the joint interface, resulting in a lower shear strength. Michael et al. [32] developed the Refractory Eutectic Assisted Bonding (REABond) technique and investigated the joining of silicon carbide-based composites using silicon-rich refractory eutectic phase compositions in the silicon–chromium, silicon–titanium, and silicon–hafnium systems. This comprised employing the silicon–hafnium (Si-Hf) eutectic phase material in tape form for joining yielded joints that were uniform, dense, well-adhered, and free of pores or voids. When using Si-Cr and Si-Ti eutectic pastes for joining, the formed joints were non-uniform, with unconnected regions, microcracks, or large gaps, as depicted in Figure 8. The shear strength of the joined specimens was related to the fiber orientation of the composites. For species with fibers parallel to the joint orientation, fractures occurred within the composites, while for specimens with fibers perpendicular to the joint orientation, fractures occurred at the joint.

## 4. Soldering Joining Technology

Soldering, a joining method that achieves atomic bonding of two or more workpieces by heating, applying pressure, or using both, holds an important position in the joining of CMCs [33].

Soldering joints can realize the connection of CMCs and possess the following advantages:

1. Superior joining strength. Under suitable process conditions, a notably high joining strength can be attained, endowing the joints with excellent mechanical properties and fulfilling the connection requirements of certain structural components with high strength demands;
2. High operating temperature tolerance. Some soldering techniques are applicable at elevated temperatures, making them suitable for joining components in high-temperature environments and conforming well to the high-temperature application scenarios of CMCs.

Nevertheless, several issues remain when employing the soldering joining method:

1. Complex process. It is necessary to precisely control multiple process parameters, such as temperature, pressure, and soldering speed. This imposes high requirements on equipment and operators, increasing production costs and operational difficulties;
2. Defect-prone joints. Soldering is prone to defects such as cracks and pores. These defects will reduce the quality and performance of the joints, affect the reliability of the joining, and hurt the properties of the materials. In addition, during the soldering process, unfavorable reactions may occur between the matrix material and the reinforcing material, leading to a decline in the performance of the reinforcements. Therefore, the soldering time and temperature generally cannot be too long or too high [34];
3. Presence of high-temperature affected zones. Soldering that requires high temperatures will cause changes in the microstructure of the CMCs, affecting their mechanical and physical properties and reducing the stability of the materials.

### 4.1. Brazing Joining Technology

**Figure 9:**
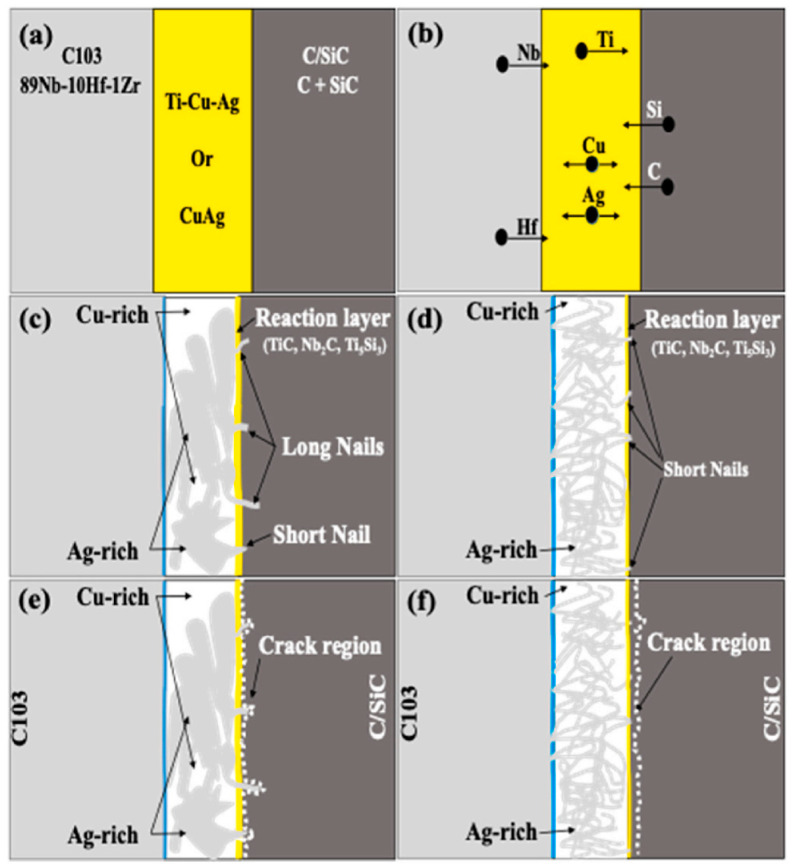
Microstructural evolution mechanisms: (**a**) before joining; (**b**) during brazing, joint based on Ticusil filler; (**c**) after brazing, joint based on Ticusil filler; (**d**) after brazing, joint based on Cusil filler; (**e**) during LSS testing, joint based on Ti-Cusil filler; (**f**) during LSS testing, joint based on Cusil filler [36].

**Figure 10:**
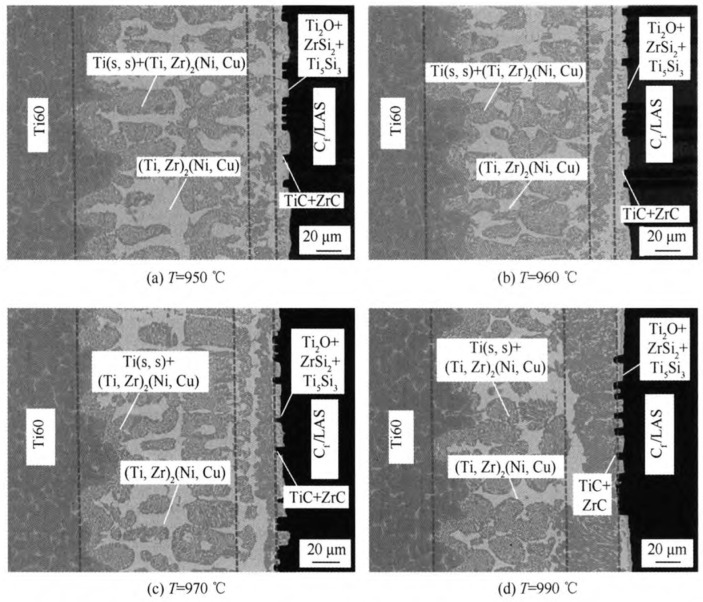
BSE images of the joint interface microstructure brazed for 10 min at different temperatures [39].

Brazing represents the most prevalently employed technique in the soldering of CMCs. It makes use of brazing filler metal, whose melting point is lower than that of the base material, as the filling medium. Upon being heated to the designated brazing temperature, the brazing filler metal liquefies and wets the base material. Subsequently, it infiltrates the joint gap via capillary action and diffuses with the base material, thereby effectuating the joining. The relatively low brazing temperature serves to preclude excessive thermal impairment to the composite material. Moreover, it exhibits a high degree of flexibility in accommodating the connection of complex geometries [35]. Anurag et al. [36] employed commercially available Ti-containing Cu-Ag alloy brazing filler metals with high activity (Ticusil) and low activity (Cusil) to successfully establish the brazing joining between C/SiC composites and niobium alloy C103. Through a detailed analysis of the microstructural characteristics of the joints and fracture surfaces, it was ascertained that the joint interface was devoid of cracks and pores, exhibiting a sound microstructure. Notably, the fracture surfaces of Ticusil-based and Cusil-based joints manifested significant disparities, with distinct crack propagation patterns. The micro-evolution mechanism is illustrated in Figure 9. Subsequently, a quadratic model was constructed via response surface methodology (RSM) analysis to prognosticate the impact of temperature, reaction time, and cooling rate on the lap shear strength of the joints. The predicted values demonstrated a high degree of congruence with the experimental ones. The strength and high-temperature resistance of brazed joints are limited by the properties of the brazing filler metals, and thus some new brazing filler metal systems are continuously being explored. Wang et al. [37] fabricated CaO-Y_2_O_3_-Al_2_O_3_-SiO_2_ (CYAS) glass and employed CYAS glass powder as the solder for joining SiC_f_/SiC composites. As the brazing temperature ascended, the intermediate layer became dense and devoid of defects, concomitantly leading to an augmentation in the joint shear strength. The optimal temperature was determined to be 1400 °C, at which point the average joint strength reached 57.1 MPa. The holding time at 1400 °C exerted a minimal influence on the intermediate layer. Extending the holding time was conducive to eradicating residual bubbles and facilitating the infiltration of the solder, with 30 min identified as the optimal holding duration. Donatella et al. [38] adopted Si-16.2Ti alloy as the brazing filler metal and accomplished the joining of the composites by the reactive infiltration process. They experimentally investigated its wettability and microstructural evolution on glassy carbon and SiC substrates, and analyzed interfacial phenomena including wettability, reactivity, and interfacial microstructural evolution, thereby furnishing a foundation for the selection of appropriate joining process parameters (temperature, time, atmosphere). Hu et al. [39] employed a Ti-Zr-Ni-Cu active alloy to braze continuous carbon fiber-reinforced lithium aluminosilicate glass–ceramic matrix composites (C_f_/LAS composites) and Ti60 alloy. It was observed that with the increase in brazing temperature, the reactants in the intermediate layer increased, and the shear strength of the joint initially increased and then decreased. By investigating the microstructure of the brazed joint interface, as shown in Figure 10, the mechanism of fracture failure was revealed at the joint. Wang et al. [40] investigated the performance of C_f_/SiC-GH3044 joints by adding diamond (C) powder to the Cu-Ni-Ti brazing filler. It was discovered that the addition of C powder could effectively enhance the mechanical properties and high-temperature resistance of the joints. The analysis of the joint’s microstructure indicated that the addition of C particles inhibited the excessive growth of the brittle compound layer and simultaneously increased the initial liquefaction temperature of the intermediate layer. The room-temperature shear strength of the joint initially increased and then decreased. When both C and Ni powders were added simultaneously, a similar trend was observed; however, a higher joining temperature and a longer holding time were required to obtain a good joint. The high-temperature shear strength of the joint generally decreased, but as the amounts of C and Ni powders added increased, the reduction in strength gradually diminished.

### 4.2. Transient Liquid Phase Joining Technology

Transient liquid phase (TLP) is a method that employs an intermediate layer alloy to form a liquid phase during heating, which promotes atomic diffusion and realizes rapid joining. The advantages of TLP joining lie in its ability to achieve high-strength joints at relatively low temperatures and within a short time, while effectively alleviating thermal stress. This is because the presence of the liquid phase accelerates the diffusion rate of atoms, making the joining process more rapid. Compared with traditional soldering methods, the temperature for TLP joining is typically 200–300 °C lower than the melting point of CMCs, and the soldering time can be shortened to several minutes to tens of minutes. Huang et al. [41] employed a Ti/Cu/Ti multilayer interlayer to join Al_2_O_3_-TiC CMCs and Q235 low-carbon steel by liquid-phase diffusion joining. The microstructure of the interface was observed using metallographic microscopy and electron probe microanalysis to analyze the elemental distribution. The results show that the TiC ceramic is tightly bonded to Q235 steel through an interface layer, with no defects present at the interface. The interface layer consists of two parts: a thin reaction layer near the TiC ceramic, which is a TiO layer, playing a key role in wettability, and a thicker layer of Cu–Ti–O and Fe–Ti–O compound layers. Element diffusion, composition changes, and the formation of the reaction layer contribute to the high integrity of the connection joint. A mechanism for the formation of the interface layer was also proposed. This study provides new directions for the application of TiC ceramic matrix composites.

### 4.3. Other Soldering Joining Technology

**Figure 11:**
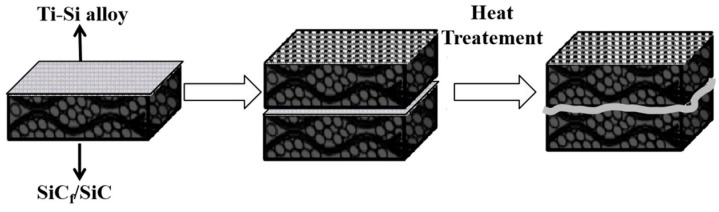
Diagram of Ti_16_Si_84_ alloy joining SiC_f_/SiC composite [42].

**Figure 12:**
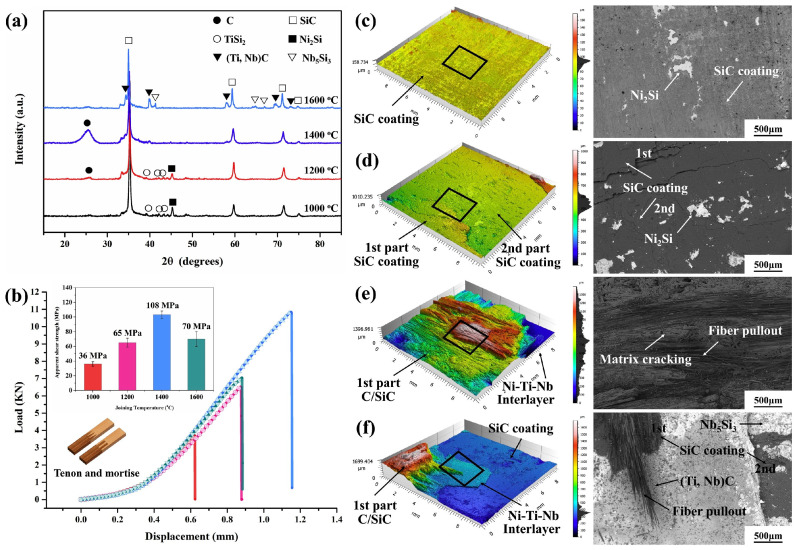
(**a**) XRD patterns of the C/SiC fracture after single-lap shear strength test; (**b**) Shear strength of C/SiC joint; 3D images and BSE images of C/SiC joint fracture: (**c**) NS1 1000 °C, (**d**) NS2 1200 °C, (**e**) NS3 1400 °C, and (**f**) NS4 1600 °C [43].

To reduce the defects in joints and lower or transfer the residual thermal stress inside the joints, researchers have developed some new methods to improve the stability of soldered joints. Xia et al. [42] fabricated a Ti_16_Si_84_ alloy for the pressureless joining of SiC_f_/SiC composites, as depicted in Figure 11. SiC_f_/SiC composites can be successfully joined at temperatures of 1390 °C and above. At 1410 °C, a smooth, dense, and defect-free joint is formed, and the shear strength of the sample reaches a maximum of 42.5 MPa at this temperature. Microstructural analysis of the joint reveals that there is no intermediate phase among SiC, TiSi_2_, and Si phases in the joint. The tight and defect-free structure and the excellent permeability of the alloy enhance the mechanical properties of the joint. After three days of hydrothermal corrosion, the remaining shear strength after corrosion is 38.37 MPa, exhibiting excellent corrosion resistance. Zhao et al. [43] devised a Ni-Ti-Nb multilayer material and successfully established a joint between the three-dimensional C/SiC composites coated with SiC and the Ni-Ti-Nb multilayer material serving as an interlayer by the spark plasma sintering (SPS) technique. Consequently, reliable joints featuring high shear strength and fewer micro-defects were acquired. Moreover, the formation of the rough joining interface was elucidated from the perspectives of interface microstructure and thermodynamics. Notably, the joining temperature exerts a pronounced influence on the mechanical properties of shear. Empirically, at 1400 °C, reliable joints with a shear strength of 108 ± 5 MPa were attained, as illustrated in Figure 12. Kolenak et al. [44] developed a novel Bi-based brazing filler metal, Bi_11_Ag_1_Mg, and successfully achieved the connection between Al_2_O_3_ ceramics and Ni-SiC composites. At the Al_2_O_3_/Bi_11_Ag_1_Mg joint interface, magnesium reacted with the ceramic substrate to form a high-magnesium reaction layer about 2 μm thick, which facilitated the bonding at the interface. At the Bi_11_Ag_1_Mg/Ni-SiC joint interface, the high silver content promoted the bonding, and high concentrations of Bi and Ni were also observed at the interface. The average shear strength of the Al_2_O_3_/Ni-SiC joint was 27 MPa. Bi-based brazing fillers have a lower melting point and good electrical conductivity, making them suitable for high-temperature and special material connections.

## 5. Hybrid Joining Technology

Hybrid joining, which is regarded as an efficacious joining modality, amalgamates the merits of diverse joining techniques, thereby proffering novel solutions for the joining of CMCs and augmenting the likelihood of their extensive application. The hybrid joining techniques pertinent to CMCs predominantly encompass the mutual combinations of mechanical joining, bonding, and soldering joining [45]. Hybrid joining, as an effective joining approach, integrates the advantages of multiple joining techniques. For instance, mechanical joining provides reliability, adhesive bonding offers sealing performance, and soldering joining ensures high strength. It can meet complex engineering requirements and enhance the comprehensive performance of the joint. According to specific application needs, different joining techniques can be selected and combined to achieve complementary performances of the joint in aspects such as strength, sealing, and high-temperature resistance, thereby optimizing the joining effect. It applies to different application scenarios and can flexibly respond to various joining requirements, enhancing the versatility of joining techniques [46]. However, it involves the operations of multiple joining techniques and the control of process parameters, necessitating the coordination and optimization of different processes and thereby increasing the complexity and difficulty of the process. The process requirements of different joining techniques may mutually constrain one another. For instance, in the hybrid joining of mechanical joining and brazing, it is essential to ensure both the firmness of mechanical joining and the excellent wetting and filling of the brazing filler metal during the brazing process, which imposes stricter requirements on the control of the process parameters. In practical operations, parameters such as soldering temperature, pressure, time, and the pre-tightening force of mechanical joining need to be precisely adjusted. Any deviation in a single parameter may impact the quality of the joint. Since multiple joining materials and equipment are required along with more complex process control, the cost of hybrid joining is relatively high. The procurement and storage of multiple joining materials increase the material cost. At the same time, the maintenance of different equipment also raises the equipment cost. Additionally, the implementation of complex processes demands more professional operators and a longer production cycle, further increasing the labor cost and time costs. For example, when adopting the hybrid joining that combines diffusion soldering and brazing, diffusion soldering equipment and brazing equipment are needed, and the parameter adjustment and maintenance of both types of equipment must be carried out by professionals, which undoubtedly increases the production cost [47].

**Figure 13:**
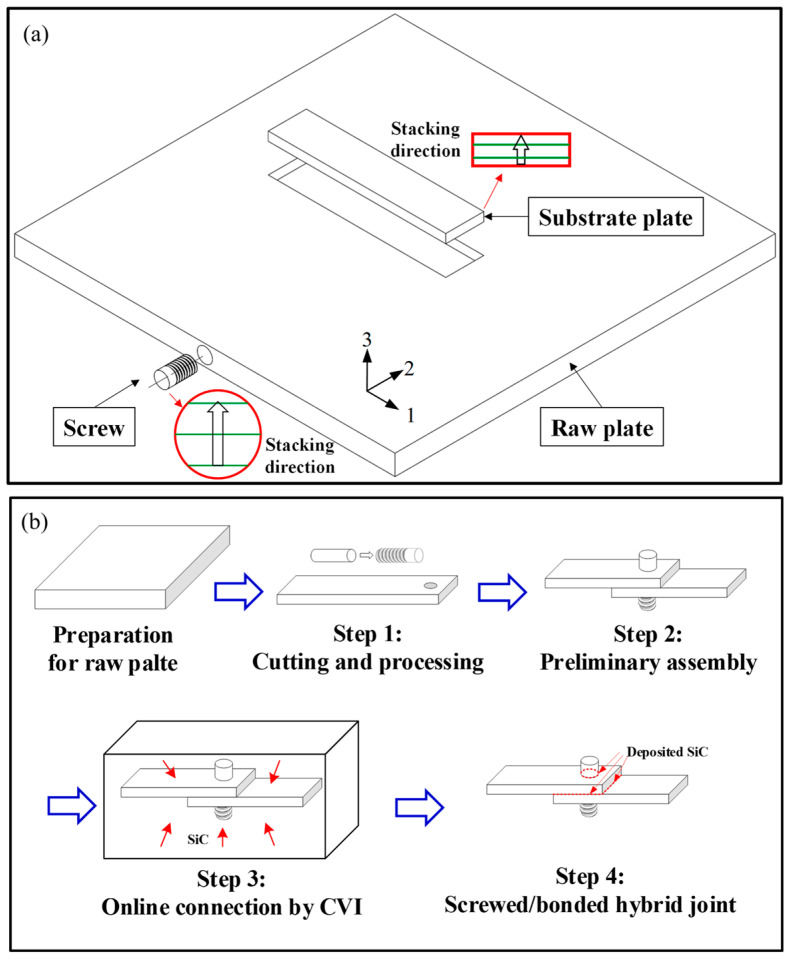
Explanation of the preparation and assembly process of the hybrid joint: (**a**) material orientation of the substrate and screw; (**b**) assembly process of the hybrid joint [48].

**Figure 14:**
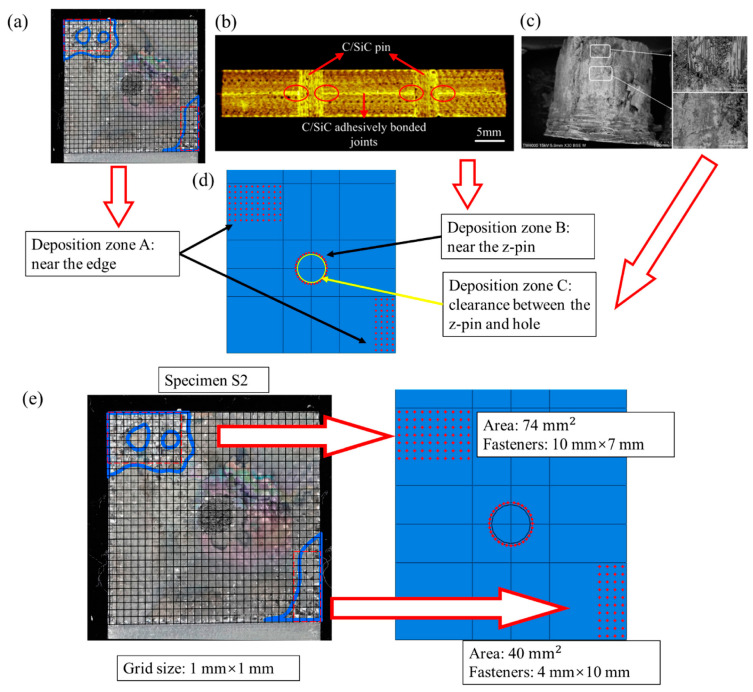
Three types of SiC deposition zones in the C/SiC z-pinned/bonded hybrid single-lap joint: (**a**) deposition zone A: near the edge; (**b**) deposition zone B: near the z-pin; (**c**) deposition zone C: gap between the z-pin and hole; (**d**) numerical model; (**e**) distribution of fasteners with similar area in deposition zone A [49].

Due to the application scenarios of CMCs, the hybrid joining of mechanical joining and bonding has been widely studied. Li et al. [48] investigated the screwed/bonded hybrid joints for two-dimensional C/SiC composites through experiments and numerical simulations. The preparation and assembly processes are depicted in Figure 13. Through tensile tests, it was found that the failure mechanisms of the two-dimensional C/SiC screw-bonded hybrid joints can be divided into two main modes: screw shear failure and substrate cracking failure. The experimental results show that during the tensile process, the deposited SiC bonding layer is the first to fail, and the load is then primarily borne by the screw, ultimately leading to either screw shear or substrate cracking. The failure process was monitored using acoustic emission (AE) and digital image correlation (DIC) technologies, and four damage modes along with their corresponding frequency ranges were identified using the K-means clustering analysis method. Numerical simulations further revealed that the uneven distribution of the SiC deposition layer causes response fluctuations during the early loading stages of the joint, while the screw assembly angle significantly affects the final failure mode and strength of the joint. This study provides important insights into the design and engineering application of C/SiC hybrid joints. Wang et al. [49] conducted research on the mechanical properties and failure mechanism of the C/SiC composites Z-pinned/bonded hybrid single-lap joints. They analyzed the impact of the distribution and strength of the secondarily deposited SiC matrix on the joint performance, as illustrated in Figure 14. In the tensile test, the deposited SiC matrix was distributed discretely, mainly concentrated at the diagonal positions of the overlapping area. The load–displacement curve of the joint showed a bimodal characteristic, with an average strength of 193.35 MPa. Upon failure, the Z-type rivet fractured, and the SiC matrix distribution was uneven. The first load peak was dominated by the edge SiC matrix, which experienced tensile failure first due to secondary bending. The second load peak was dominated by the Z-type rivet and the nearby SiC matrix. The Z-type rivet initially experienced failure in the transverse fibers, followed by fracture, and the nearby SiC matrix also failed. The effects of four distribution patterns of the deposited SiC matrix in the edge area were discussed. The joint strength was the highest in the transverse pattern and the lowest in the longitudinal pattern Li et al. [50] employed in situ micro-CT technology to analyze the damage evolution of screwed/bonded hybrid joints of C/SiC under tensile loads. The SiC adhesive layer was densely distributed at both ends along the length direction of the bolt assembly but sparse in the middle. The size of the prefabricated bottom hole influenced the initial assembly tightness, which interacted with the bottleneck effect of the CVI process on the assembly clearance. In the early stage, when the bolt carried the load alone, there were mainly two types of failures: debonding failure of the SiC layer deposited in the assembly clearance of the bolt hole, and local shear failure of the thread teeth. In the substrate overlap region, interlaminar shear failure occurs at areas with dense SiC deposition, accompanied by fiber shear and pull-out.

## 6. Analysis on the Development Trends of Joining Technologies

Based on the research conducted by the aforementioned researchers, the shear strength, failure mode, and temperature resistance of different joining structures for ceramic matrix composites can be obtained, as shown in Table 1. The mechanical joining, bonding, soldering, and hybrid joining techniques of CMCs each have their unique advantages and disadvantages, as shown in Table 2, and are suitable for different scenarios. Soldering joining has potential in high-temperature applications and high-strength bonding, but the process is complex and prone to defects. Mechanical joining is highly reliable and easy to operate, but it can increase mass and cost. Bonding joining offers good sealing performance and is simple to operate, but the joint strength and application temperature are limited. Hybrid joining combines the advantages of multiple technologies, but the process is complex and the cost is relatively high [51]. In practical applications, the most appropriate joining technique or combination of techniques should be comprehensively considered and selected based on specific engineering requirements, material characteristics, operating environments, costs, and other factors to achieve the optimal performance and reliability of ceramic matrix composite structures.

The joining technology of CMCs is developing towards optimizing process parameters, developing new joining materials, innovating joining methods, and achieving the integration of multiple technologies. For example, improving joint quality and performance through precise control of process parameters; exploring new intermediate layer materials or brazing materials to meet a broader range of application needs; researching new joining mechanisms and processes, such as in situ reactive bonding; and combining the advantages of multiple joining technologies to develop composite joining processes.

Currently, many researchers are working on new connection technologies for ceramic matrix composites, providing more possibilities for their applications, especially in extreme environments and high-performance requirements. Zhou et al. [52] were the first to successfully achieve the seamless bonding of SiC_w_/Ti_3_SiC_2_ composites at 1090 °C in just 30 s using electric field-assisted sintering technology (FAST) without any additional materials. Solid-state diffusion was the primary mechanism, and high current density facilitated the process. The average shear strength of the 1090 °C bonded samples reached 51.8 ± 2.9 MPa, with failure occurring in the matrix, ensuring reliable bonding. Other technologies such as ultrasonic welding [44] and spark plasma sintering (SPS) [53] have also been explored. These technologies typically offer unique advantages, such as rapid bonding, low thermal impact, and high precision, but also face challenges like high costs and complex processes. With ongoing advancements in materials science and manufacturing technologies, these emerging connection techniques are expected to see broader applications and optimization in the future.

In the future, breakthroughs should be made in the study of joining mechanisms to gain a deeper understanding of atomic-scale interface reactions, diffusion processes, and stress evolution mechanisms, providing a theoretical basis for the precise control of the joining process. Using advanced characterization techniques and computational simulation methods, the microscopic behavior of different materials during the joining process should be revealed, including atomic diffusion pathways and the formation process of interface reaction products. This will help optimize joining process parameters, improve the predictability of joint performance, achieve precise control of the joining process, and ensure the stability and reliability of joint quality.

Developing high-performance joining materials is one of the key directions. Research and development of brazing materials, intermediate layer materials, and other materials with better wettability, higher strength, and improved resistance to high temperatures and corrosion are essential to meet the joining requirements of CMCs under extreme conditions and achieve superior joining performance.

With the development of intelligent manufacturing technologies, intelligent control of the joining process can be achieved. By real-time monitoring of parameters such as temperature, pressure, and stress during the joining process, and using artificial intelligence algorithms for data analysis and feedback control, the automation and precision of the joining process can be realized, improving the stability and consistency of connection quality.

Interdisciplinary research will bring new opportunities to the joining technology of CMCs. By integrating knowledge from materials science, physics, chemistry, mechanical engineering, and other disciplines, innovative joining methods and technologies can be developed to address the challenges faced by existing joining technologies, such as thermal stress issues, interface compatibility problems, and more. This will promote the comprehensive development of CMC-joining technologies and enable broader engineering applications.

## 7. Failure Behavior and Performance Prediction of CMCs Joining Structures

### 7.1. Failure Behavior and Evaluation Methods

The failure behavior at joints varies with different joining methods. In mechanical joining, due to issues with bolt quality and strength, fractures are prone to occur; poor hole size accuracy and surface roughness can lead to poor bolt fit and loosening. Additionally, excessive bolt tightening torque can cause matrix cracking, while insufficient torque may fail to ensure proper fastening. Rivet strength may be inadequate, leading to shearing under load; gaps between the rivet and the connected part may result in a pullout from the hole. Stress concentration around the joining hole can also trigger matrix cracking, reducing the joining strength. In bonding, issues such as adhesive layer quality, the bonding process, and environmental factors can lead to adhesive delamination; insufficient adhesive strength or incomplete curing may reduce cohesive strength, resulting in cohesive failure [54]. In soldering joining, solder defects or residual stresses generated during the soldering cooling process can lead to weld cracking. The thermal cycle from soldering creates a heat-affected zone (HAZ) around the weld, where material properties change, with increased hardness and reduced toughness, making it more brittle and prone to cracking, thus affecting the joint strength.

There are various methods and perspectives to evaluate the failure behavior of CMC-joining structures. Non-destructive testing (NDT) evaluation includes ultrasonic testing, radiographic testing, CT scanning, and infrared thermography. These techniques analyze the changes in physical signals as they propagate through the tested object to identify defects in terms of size, location, and shape. Mechanical performance testing evaluation includes tensile tests, shear tests, fatigue tests, and impact tests. These tests assess load-bearing capacity and failure behavior by measuring mechanical performance indicators and analyzing load–displacement curves. Microstructural analysis evaluation uses optical microscopes, scanning electron microscopes (SEM), and transmission electron microscopes (TEM) to observe the microstructure and gain deeper insights into the failure mechanisms and damage evolution of the joining structure. Numerical simulation and analysis evaluation involve finite element analysis (FEA) and multiscale simulations to predict stress distribution and potential failure locations, optimize design and process parameters, and more accurately describe the mechanical behavior and failure process of the connection structure [55].

However, traditional non-destructive testing methods, while capable of providing some information about microcracks, cannot comprehensively and intuitively capture the morphology and quantitative information of the cracks. Moreover, existing image segmentation methods, such as grayscale thresholding and region-growing algorithms, have limitations when dealing with low grayscale contrast and noise interference, making it difficult to accurately segment tiny cracks [56].

In recent years, with the rise in artificial intelligence, deep learning methods have made significant progress in the field of computer vision, especially in image segmentation. Zhu et al. [57] proposed a deep learning-based microcrack segmentation method using generative adversarial networks (GANs), combined with in situ X-ray microtomography (μCT), to quantitatively characterize the microcrack evolution behavior of CMCs under high-temperature tensile loading. This method can accurately and robustly segment microcracks with low grayscale contrast and low quality due to noise and artifacts in μCT images, and it can capture parameters such as crack-opening area, crack-opening displacement, and crack volume. The approach is not only applicable to CMCs, but also to the study of microcracks in ceramic matrix composite connection structures, particularly in dynamic damage mechanism studies, providing a reliable solution for capturing microcrack information from low-quality images.

### 7.2. Performance Prediction Methods

Predicting the performance of CMC-joining structures is of significant importance, with the following four main aspects:

1. Ensure safety and reliability in critical applications such as aerospace and energy, avoiding serious accidents caused by joining failure;
2. Allows for design optimization by using methods such as numerical simulation to analyze the impact of design parameters, determining the optimal material combinations and joining processes, reducing stress concentration, and improving load-bearing capacity;
3. Performance prediction helps to assess service life and simulate the effects of fatigue, creep, oxidation, and other factors to determine fatigue life and rationalize maintenance and replacement schedules;
4. Reduces costs, reduces the number of tests, improves R&D efficiency, and avoids additional costly expenditures due to the failure of the joining structure.

**Figure 15:**
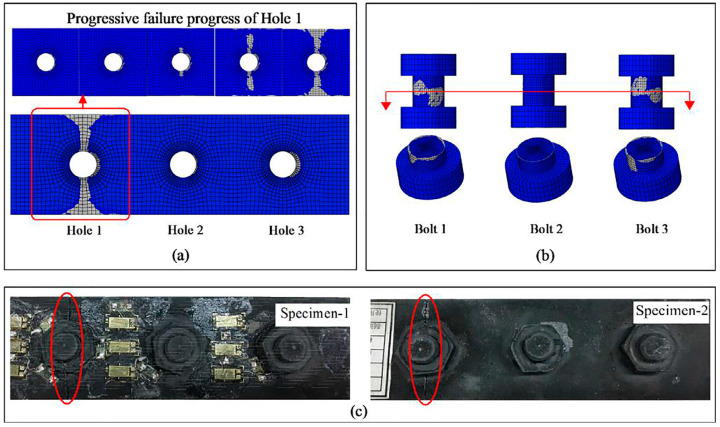
Failure comparison between numerical simulation and experimental results: (**a**) numerical simulation failure mode and predicted progressive failure process; (**b**) numerical simulation failure diagram of the bolt; (**c**) typical failure modes in the experiment [58].

**Figure 16:**
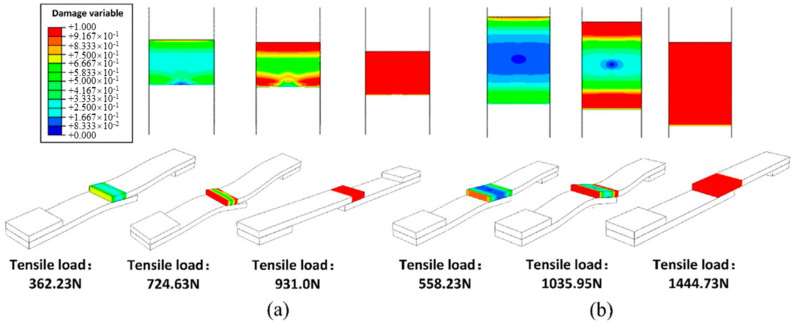
Simulation of the failure process: (**a**) SiC bonding layer in the semi-lap region; (**b**) SiC bonding layer in the entire lap region [60].

The performance prediction of the CMC-joining structure is mainly carried out using finite element analysis simulation. Wang et al. [58] proposed a progressive damage model (PDM) to analyze the failure of fully C/SiC composite multi-bolt joints. Through progressive damage analysis of C/SiC composite perforated laminates and comparison with experimental results, as shown in Figure 15, they determined a combined degradation model to establish the PDM for C/SiC composite structures. The failure strength and modes of the joints were predicted and validated through experiments. Vandellos et al. [59] studied the mechanical behavior of woven ceramic matrix composite adhesive joints under four-point bending tests through experimental and numerical analysis. An ONERA Damage Model (ODM) was used to model the adhesive joints of woven ceramic matrix composite plates. The numerical simulation results showed a good correlation with the failure behavior observed in the experimental samples, and the model can be used to analyze the damage mechanisms and predict failure behavior at the joining site. The author [60,61] analyzed the tensile behavior and failure mechanisms of two-dimensional C/SiC composite Z-pinned/bonded hybrid single-lap joints through experimental and numerical simulation methods. An improved shell-fastener finite element model was established, and the numerical simulation results showed good overall agreement with the experimental results. The pin diameter was found to be related to the ultimate shear strength value. By establishing contact models for different bonding regions, it was demonstrated that the SiC bonding layer could enhance the shear strength of the joint. The simulation results are shown in Figure 16.

Although finite element analysis (FEA) is a widely used numerical simulation method for predicting the mechanical properties, stress distribution, deformation behavior, and other characteristics of materials or structures, it also has some limitations, especially when comparing with experimental results, where potential discrepancies may arise. These discrepancies mainly stem from the following aspects:

1. Model simplifications and assumptions: Finite element analysis often requires simplifying complex real-world structures, neglecting certain microstructural features and material defects. These simplifications may lead to the model not fully capturing the complex behavior of the actual material;
2. Limitations of material constitutive models: Finite element analysis relies on material constitutive models (such as elasticity, plasticity, creep, etc.), which are often based on idealized assumptions. If the actual behavior of the material deviates from the assumptions of the model, the prediction results may be inaccurate;
3. Uncertainty in boundary conditions and loading: Finite element analysis requires precise boundary conditions and load inputs. However, in actual experiments, the loading conditions are often complex, and boundary conditions and loads are often difficult to fully control or measure;
4. Numerical errors: The accuracy of finite element analysis depends on the refinement of the mesh. A mesh that is too coarse may lead to numerical errors, while an excessively fine mesh increases computational costs;
5. Impact of microstructure and defects: Finite element analysis is typically conducted at the macroscopic scale, making it difficult to fully account for the influence of the material’s microstructure (such as grains, pores, cracks, etc.) on its overall performance.

To reduce these discrepancies, it is generally necessary to calibrate and validate the finite element model using experimental data, and to combine multi-scale simulation methods (such as molecular dynamics, micro-mechanics, etc.) to improve the accuracy of predictions.

With the continuous development of the Internet of Things (IoT), big data, and artificial intelligence technologies, the application of digital twins [62] in industrial performance prediction is becoming widespread and more in-depth. By introducing digital twin technology into the performance prediction of ceramic matrix composite joining structures, high-precision performance predictions and optimization solutions can be provided through the integration of real-time data, physical models, and artificial intelligence techniques. This can significantly enhance the efficiency, reliability, and safety of industrial systems while reducing operational costs and risks, offering broad prospects for industrial applications of ceramic matrix composites.

## 8. Conclusions and Application Perspectives

This paper presents the research progress on CMC-joining structures and analyzes their future development trends. Although CMC-joining structures have been extensively studied by researchers, there are still several issues that need to be addressed:

1. CMCs suffer from the problem of interfacial reactions during joining, especially when joining with dissimilar materials (e.g., metals). Effective interfacial modification methods should be developed to improve interfacial stability and bond strength;
2. In practical applications, there are still issues with the stability of CMC-joining processes. Further optimization of joining processes is needed to ensure performance consistency of the joining structure under various working conditions;
3. Exploration and development of new CMC-joining processes. Emerging additive manufacturing technologies offer the potential for integrated manufacturing and the joining of complex structures. Although many challenges remain, these technologies have promising application prospects and may overcome the limitations of traditional joining processes.

CMCs have broad application prospects in fields such as the aerospace, energy, and automotive industries, and their joining structures demonstrate immense potential for future applications. In the aerospace field, reliable joining technologies can ensure the performance and reliability of engine hot-end components, thermal protection structures, and other parts under high-temperature, high-pressure, and complex stress environments, driving the development of aerospace technology. In the energy sector, the development of joining technologies helps to improve the performance and safety of nuclear reactors, solar energy systems, and other devices, meeting the demands for high temperature, corrosion resistance, and radiation resistance in materials. In the automotive industry, joining technologies enable effective connections between CMCs and other automotive component materials, playing a key role in vehicle manufacturing. This can facilitate the efficient joining of lightweight structures, enhancing vehicle performance and fuel efficiency [63].

However, the widespread application of ceramic matrix composite joining structures in industrial production still faces numerous challenges:

1. Cost-effectiveness: CMCs are often composed of high-performance ceramic matrices and reinforcement materials, which are typically expensive. For example, in silicon carbide ceramic matrix composites, materials such as silicon carbide powder and carbon fibers have high costs for obtaining high purity and quality. To achieve the connection of ceramic matrix composites, special bonding materials and advanced connection equipment are often required. For instance, when using active brazing for bonding, the active brazing materials are costly; laser joining technology requires the purchase of high-power, high-precision laser equipment. Some ceramic matrix composite joining technologies have relatively low production efficiency. For example, diffusion bonding requires long holding times and precise temperature control, making the process slow and difficult to meet the demands of large-scale, rapid production. Additionally, the process often has a low yield rate, which leads to a large amount of material waste and extra production costs;
2. Process control: The joining of CMCs often requires the coordination of multiple parameters, such as temperature, pressure, time, and atmosphere. Strict parameter requirements mean that even small fluctuations in parameters can affect the quality of the final product. Additionally, different material systems and joining processes require different parameter combinations. In large-scale production, managing a variety of product models and material specifications, finding the appropriate parameter matches, and quickly switching between them, presents a high demand for process control;
3. Quality assurance: Factors such as batch variations in raw materials, fluctuations in equipment performance, and differences in operator skill levels can all affect the quality of the joining process. How to implement a strict quality control system to monitor and manage the entire production process, ensuring consistent product quality, is a key issue in industrial manufacturing;
4. Standards and regulations: Currently, the application of ceramic matrix composite joining technology in the industrial field is still in the development stage, and there is a lack of unified and comprehensive industry standards and regulations. Different companies may adopt different joining processes and quality control methods, leading to varying product quality and making it difficult to form a unified quality recognition in the market, hindering large-scale adoption. The testing and evaluation methods for the joining quality of ceramic matrix composites are still not mature and comprehensive. Existing testing methods may not fully and accurately reflect the actual performance and reliability of the joint, especially for connection performance testing under complex operating conditions, where there is a lack of effective simulation and evaluation methods. This creates difficulties in product quality control and certification. The defects in ceramic matrix composite joints are diverse, with some being very small and difficult to identify using conventional methods. Moreover, different types and sizes of defects can have varying impacts on product performance. Accurately identifying and assessing the severity of defects, and establishing reasonable quality standards, are challenges in quality assurance;
5. Environmental and sustainability issues: During the joining process of ceramic matrix composites, certain chemicals are often used, such as flux in some active brazing processes and acid–base solutions for surface treatments. Many of these chemicals are toxic and corrosive. For example, fluxes containing elements like fluorine and chlorine may release harmful gases during use, posing health risks to operators. If discharged untreated, they can pollute the soil, water bodies, and the environment, impacting the ecological balance. In addition to harmful gases generated by chemicals, the joining process itself may produce exhaust gases. For instance, during high-temperature joining, impurities and additives in materials may volatilize or undergo chemical reactions, generating harmful gases. Organic binders in some ceramic matrix composites, for example, may decompose at high temperatures, releasing carbon dioxide, carbon monoxide, volatile organic compounds (VOCs), and other emissions. If these exhaust gases are not properly treated and purified, and are directly released into the atmosphere, they can contribute to air pollution, exacerbate the greenhouse effect, and cause photochemical smog and other environmental issues.

In-depth research into joining technologies will help overcome existing limitations, fully unleash the potential of CMCs, and promote technological advancements and sustainable development across various industries.

## Figures and Tables

**Table 1:** The shear strength, failure mode, and temperature resistance of different joining technologies.

Joining Technology		Shear Strength (Room Temperature)	Failure Mode	Temperature Resistance
Mechanical joining	Bolt joining [13,14,19]	≥100 MPa	Compressive delamination failure near the hole edge of CMCs	As the temperature increases, the limit load first decreases and then increases, reaching its minimum at 300 °C.
	Rivet joining [15,16,18,22]	≥45 MPa	The riveted area undergoes bending deformation due to the bending moment, causing the rivets to tilt and become loose.	At 800 °C, the limit load is 78.7% of the value at room temperature.
Bonding [26,27,29,30]		16 MPa~40 MPa	Debonding between the matrix and the adhesive or interlaminar delamination within the ceramic composite.	The joint is expected to withstand operating temperatures above 850 °C and harsh conditions such as combustion environments.
Soldering joining	Brazing joining [37,38,39,40]	71 MPa~250 MPa	Fracture occurs at the interface between the ceramic matrix composite substrate and the welding layer or cracking occurs inside the ceramic matrix composite and delamination occurs along the interlayers.	The maximum temperature the joint can withstand is over 1000 °C.
	Other soldering joining [42,43,44]	42 MPa~110 MPa		–
Hybrid joining [48,49,50]		100 MPa~195 MPa	The adhesive layer fails first and then causes the interlaminar shear failure of the ceramic matrix composite.	–

**Table 2:** Advantages and disadvantages of various joining technologies.

Joining Technology	Advantages	Disadvantages
Mechanical joining	High joint strength; High reliability; Easy to disassemble	Increase in mass; Damage to the joined components; High cost
Bonding	Reliable sealing; Low preparation temperature; Simple operation	Low joint strength; Limited application temperature
Soldering joining	High joining strength; High application temperature; Relatively mature technology	High permeability; High cost; Residual stresses in joints
Hybrid joining	High joining strength; High reliability; High adaptability	Complex process; High cost

## Data Availability

No new data were created or analyzed in this study.
